# Prognostic Model and Nomogram Construction Based on a Novel Ferroptosis-Related Gene Signature in Lower-Grade Glioma

**DOI:** 10.3389/fgene.2021.753680

**Published:** 2021-11-08

**Authors:** Junsheng Zhao, Zhengtao Liu, Xiaoping Zheng, Hainv Gao, Lanjuan Li

**Affiliations:** ^1^ State Key Laboratory for Diagnosis and Treatment of Infectious Diseases, National Clinical Research Center for Infectious Diseases, Collaborative Innovation Center for Diagnosis and Treatment of Infectious Diseases, The First Affiliated Hospital, Zhejiang University School of Medicine, Hangzhou, China; ^2^ Division of Hepatobiliary and Pancreatic Surgery, Department of Surgery, The First Affiliated Hospital, Zhejiang University School of Medicine, Hangzhou, China; ^3^ Department of Pathology, Hangzhou Tongchuang Medical Laboratory, Hangzhou, China; ^4^ Department of Infectious Diseases, ShuLan (Hangzhou) Hospital Affiliated to Zhejiang Shuren University, Shulan International Medical College, Hangzhou, China

**Keywords:** ferroptosis, lower-grade glioma, prognostic signature, TCGA, CGGA, immune statuses

## Abstract

**Background:** Low-grade glioma (LGG) is considered a fatal disease for young adults, with overall survival widely ranging from 1 to 15 years depending on histopathologic and molecular subtypes. As a novel type of programmed cell death, ferroptosis was reported to be involved in tumorigenesis and development, which has been intensively studied in recent years.

**Methods:** For the discovery cohort, data from The Cancer Genome Atlas (TCGA) and Genotype-Tissue Expression (GTEx) were used to identify the differentially expressed and prognostic ferroptosis-related genes (FRGs). The least absolute shrinkage and selection operator (LASSO) and multivariate Cox were used to establish a prognostic signature with the above-selected FRGs. Then, the signature was developed and validated in TCGA and Chinese Glioma Genome Atlas (CGGA) databases. By combining clinicopathological features and the FRG signature, a nomogram was established to predict individuals’ one-, three-, and five-year survival probability, and its predictive performance was evaluated by Harrell’s concordance index (C-index) and calibration curves. Enrichment analysis was performed to explore the signaling pathways regulated by the signature.

**Results:** A novel risk signature contains seven FRGs that were constructed and were used to divide patients into two groups. Kaplan–Meier (K−M) survival curve and receiver-operating characteristic (ROC) curve analyses confirmed the prognostic performance of the risk model, followed by external validation based on data from the CGGA. The nomogram based on the risk signature and clinical traits was validated to perform well for predicting the survival rate of LGG. Finally, functional analysis revealed that the immune statuses were different between the two risk groups, which might help explain the underlying mechanisms of ferroptosis in LGG.

**Conclusion:** In conclusion, this study constructed a novel and robust seven-FRG signature and established a prognostic nomogram for LGG survival prediction.

## Background

Lower-grade glioma (LGG) comprises of World Health Organization (WHO) grade II (diffuse lower-grade) and III (intermediate-grade) glioma tumors. Despite relatively more favorable clinical outcomes for LGG than the grade IV tumors, the survival of LGG patients widely varies from 1 to 15 years, and 70% of patients from the lower grade progress to high grade within 10 years (van den Bent, 2014). In the past decades, the histological classification has been the standard clinical approach for LGG, but it lacks the prediction of grades due to the wide range of clinical heterogeneities of LGG (Louis et al., 2016). Although some molecular markers, such as the presence or absence of mutation in isocitrate dehydrogenase (*IDH*) genes, have been integrated in the WHO classification for LGG, to identify novel prognostic biomarkers remains imperative.

Ferroptosis is a kind of programmed non-apoptotic cell death caused by an iron-dependent lethal accumulation of lipid peroxidation. As a promising therapeutic alternative, ferroptosis was reported to be closely related to multiple diseases such as neurodegenerative diseases (Do Van et al., 2016) and cancer (Shen et al., 2018). Aberrant expressions of ferroptosis-related genes (FRGs), such as tumor protein p53 (*TP53*) (Junttila and Evan, 2009), Fanconi anemia complementation group D2 (*FANCD2*) (Han et al., 2017), glutathione peroxidase 4 (*GPX4*) (Liu H. et al., 2018), heat shock protein beta 1 (*HSPB1*) (Arrigo and Gibert, 2012), and dipeptidyl-peptidase-4 (*DPP4*) (Enz et al., 2019), were reported to be correlated with tumor genesis and progression. A ferroptosis inducer, named erastin, could inhibit the tumor growth and enhance the sensitivity of chemotherapeutic drugs (Chen et al., 2015; Yu et al., 2015). Meanwhile, the induction of ferroptosis synergistically enhanced the antitumor activity of immune checkpoint inhibitors (ICIs), even in ICI-resistant tumors (Badgley et al., 2020). Pseudolaric acid B (PAB) triggered the ferroptosis and inhibited the viabilities of glioma cells (Wang Z. et al., 2018). These studies indicated the activation or inhibition of ferroptosis has potential clinical value, and better understanding of ferroptosis might provide prognostic value and therapeutic candidates for management of LGG.

**FIGURE 1:**
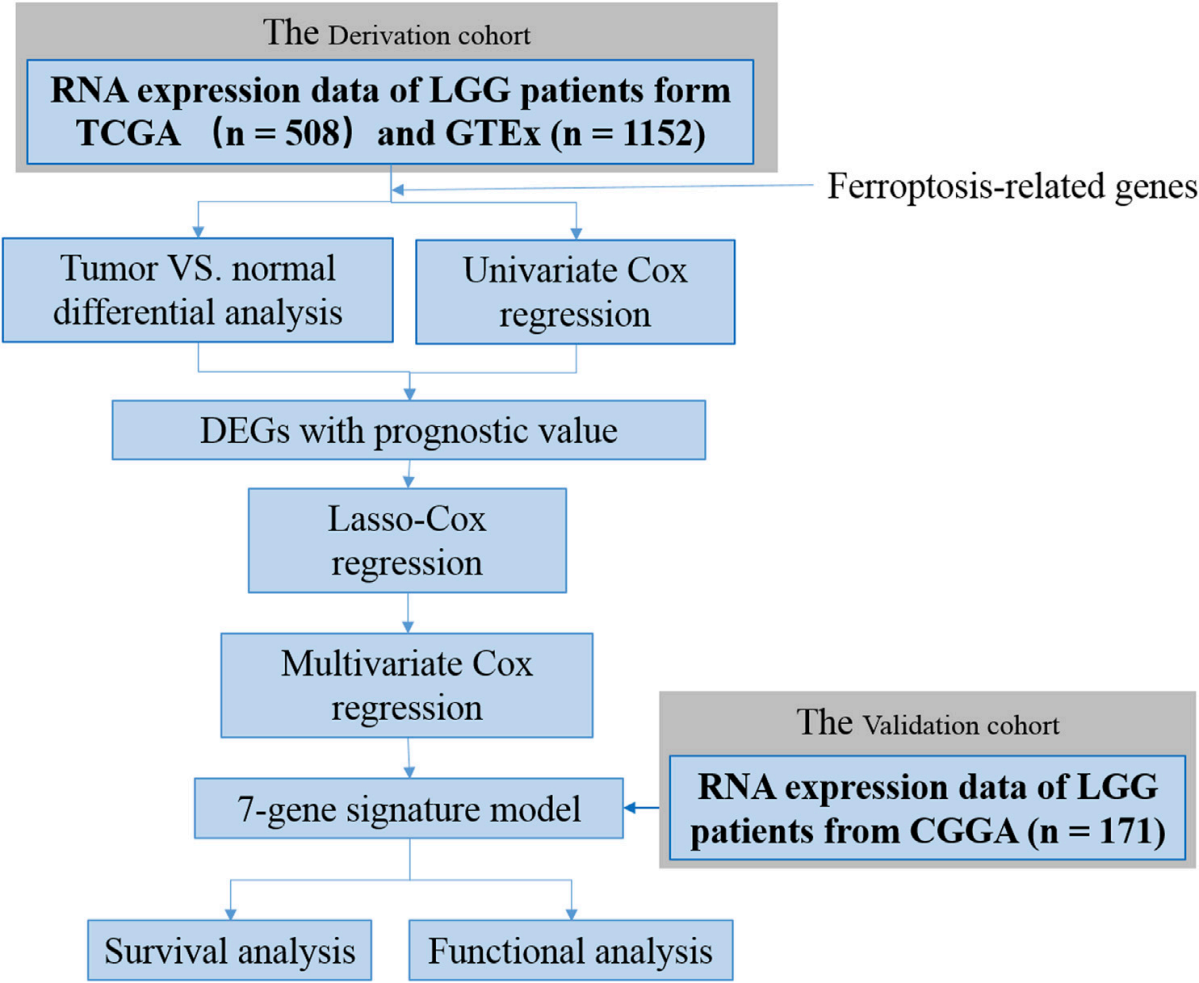
Flow chart of data collection and analysis.

In the present study, we analyzed the aberrant expression of FRGs by analyzing the LGG samples from The Cancer Genome Atlas (TCGA) and normal brain tissue from the Genotype-Tissue Expression (GTEx) database. The overall process is shown in Figure 1. To be specific, a prognostic signature was established based on seven FRGs’ expression levels which dissected the patients with LGG into two risk groups. Meanwhile, its predictive performance was elucidated by Kaplan–Meier (K–M) survival and receiver-operating characteristic (ROC) curves and further validated by two external cohorts consisting of LGG patients from the Chinese Glioma Genome Atlas (CGGA) database (Zhao et al., 2017). Furthermore, a survival nomogram was developed by integrating the risk signature with other four clinicopathological factors, and its predicted accuracy in LGG patients was assessed. Finally, enrichment analyses were performed to investigate the underlying mechanisms between the signature and tumor immunology. Overall, our data suggested that FRGs play pivotal roles in glioma progression and could be prognostic markers and therapeutic targets for glioma. The FRG signature constructed in this study might enhance the ability to predict the prognosis of LGG patients and provide potential explanations on the underlying mechanisms of ferroptosis in LGG progression.

## Methods

### Dataset and Source

MRNA expression data, DNA methylation 450K data, and corresponding clinical information of 529 LGG patients were obtained from TCGA data portal (https://gdc-portal.nci.nih.gov/). For differentially expressed gene analysis, gene expression data in normal tissue of Genotype-Tissue Expression (GTEx) were downloaded from the UCSC Xena browser (Goldman et al., 2014; Gentles et al., 2015). RNA-seq data and clinical information of other LGG sets were obtained from the Chinese Glioma Genome Atlas (CGGA) (http://www.cgga.org.cn/) (Bao et al., 2014; Wang et al., 2015; Zhao et al., 2017; Liu X. et al., 2018). Gene expression values of fragments per kilobase of exon per million reads mapped (FPKM) were normalized and subjected to the following analyses. A comprehensive list of ferroptosis-related genes (FRGs) was chosen and is provided in Supplementary Table S1.

### Development and Evaluation of the Ferroptosis-Related Prognostic Signature

To identify differentially expressed FRGs (DE-FRGs) between TCGA and GTEx databases, the absolute value of the log_2_-transformed fold change (log_2_FC) > 2 and the false discovery rate (FDR) < 0.05 was considered to be statistically significant (Zhang et al., 2018). To investigate the mechanisms of dysregulation for FRGs in LGG, Pearson’s correlation between FRGs’ transcriptional expression levels and promotor methylation levels was calculated, and those with |R| > 0.3 and *p* < 0.05 were considered significantly correlated (Xu et al., 2019).

To screen genes for constructing a risk signature, univariate Cox regression models (*p* < 0.05) were performed to select genes that are associated with the overall survival of LGG patients in TCGA. The overlapped candidate prognostic DE-FRGs were visualized in a Venn diagram. A protein–protein interaction (PPI) network for the prognostic DE-FRGs was generated by the STRING database (version 11.0) (Szklarczyk et al., 2010).

Subsequently, the least absolute shrinkage and selection operator (LASSO) Cox regression was performed by using the “glmnet” R package to shrink the scope of gene screening. The multivariate Cox regression analysis was carried out to identify highly correlated genes and construct the survival risk score model. The risk score was calculated as follows:

$$\mathrm{Risk}\;\mathrm{score=}\overset{n}{\underset{i=1}{\sum }}\mathrm{expi}\times \mathrm{\beta i},$$

where *n*, *expi*, and *βi* represent the number of genes, the expression level, and the coefficient of the gene *i*, respectively. According to the median value of the risk scores, the LGG patients were classified into low- and high-risk groups. Principal component analysis (PCA) was carried out to explore the distribution of different groups using the “prcomp” function in R. The Kaplan–Meier (K–M) curves were used to evaluate the statistical significance of the survival rates between different risk groups. The receiver-operating characteristic (ROC) curve analyses were executed to calculate the area under the curve (AUC) and to determine the predictive ability of the risk score signature. We calculated the false negative rate (FNR) of the survival prognosis by our FRG model.

In order to study whether the FRG-based prognostic signature could be used as an independent predictor for LGG patients, univariate and multivariate Cox regression analyses were conducted in TCGA and CGGA datasets. The risk score, age, gender, grade, and IDH status were used as covariates.

### Construction and Evaluation of a Predictive Nomogram

To establish a clinically applicable method for predicting the prognosis of LGG patients, we formulated a prognostic nomogram to predict the survival probability at 1, 3, and 5 years based on TCGA dataset. Five independent prognostic parameters, including age, gender, grade, IDH1 status, and risk score, were used to establish the nomogram for predicting one-, three-, and five-year survival rates of LGG patients in the “rms,” “foreign,” and “survival” R packages. Bootstraps with 1,000 resamples were used for these activities. The performance of the prognostic nomogram was assessed by calculating Harrell’s concordance index (C-index) (Harrell et al., 1996), and calibration curves of the nomogram for one-, three-, and five-year overall survival were carried out to estimate the accuracy of actual observed rates with the predicted survival probability. During the external validation of the nomogram, the total points of each patient in the CGGA cohort were calculated according to the established nomogram, and then the C-index was calculated and calibration curves were derived.

### Functional Annotation and Enrichment Analysis

The “clusterProfiler,” “org.Hs.eg.db,” “enrichplot,” and “ggplot2” packages in R were used to perform Gene Ontology (GO) and Kyoto Encyclopedia of Genes and Genomes (KEGG) annotation enrichment analyses based on the differentially expressed genes (|log_2_FC| ≥ 2, FDR <0.05) between the low- and high-risk groups. The top 30 GO terms and KEGG pathways were identified with a cutoff of *p* < 0.05 and FDR < 0.05.

The single-sample gene set enrichment analysis (ssGSEA) was conducted using the “GSVA” package in R to evaluate the enrichment score (ES) of immune parameters, including 15 immune cells and 13 immune-related pathways (Goldman et al., 2014). The annotated gene set file is provided in Supplementary Table S2. Pearson’s correlation coefficients were calculated to test the correlation between the ES and the risk score.

### Statistical Analysis

All statistical analyses were performed using R (version 4.0.3). Differences between two sample groups were tested by the Wilcoxon test. Univariate and multivariate Cox regression analyses were performed to determine risk factors for low and high risk scores of the signature in LGG. The Venn diagrams, heatmaps, boxplots, forest plots, nomograms, and calibration plots were drawn using R language. The K–M curve used the log-rank test to evaluate the statistical difference of survival rates between different risk groups. The significance of survival time differences was calculated using the log-rank test with a threshold of *p* < 0.05.

## Results

### Characteristics of LGG Patients

The workflow of this study is shown in Figure 1. A total of 508 LGG patients from TCGA-LGG cohort and 171 LGG patients from the CGGA cohort were finally enrolled. The clinical characteristics of patients from two databases are summarized in Table 1.

**TABLE 1:** Summary of clinical characteristics of the lower-grade glioma datasets.

	TCGA-LGG dataset (508)	CGGA-LGG dataset (*n* = 171)
Age (years)		
<=40	251	95
>40	257	76
Gender		
Male	282	105
Female	226	66
WHO grade		
Grade Ⅱ	246	97
Grade Ⅲ	261	74
Unknown	1	
IDH1 status		
Wild-type	290	44
Mutation	127	127
Unknown	91	
Vital status		
Alive	399	81
Dead	109	90
Survival time (days, mean ± SD)	859.5 ± 929.4	2,143.3 ± 1,542.6

### Aberrant Expression of FRGs in LGG

**FIGURE 2:**
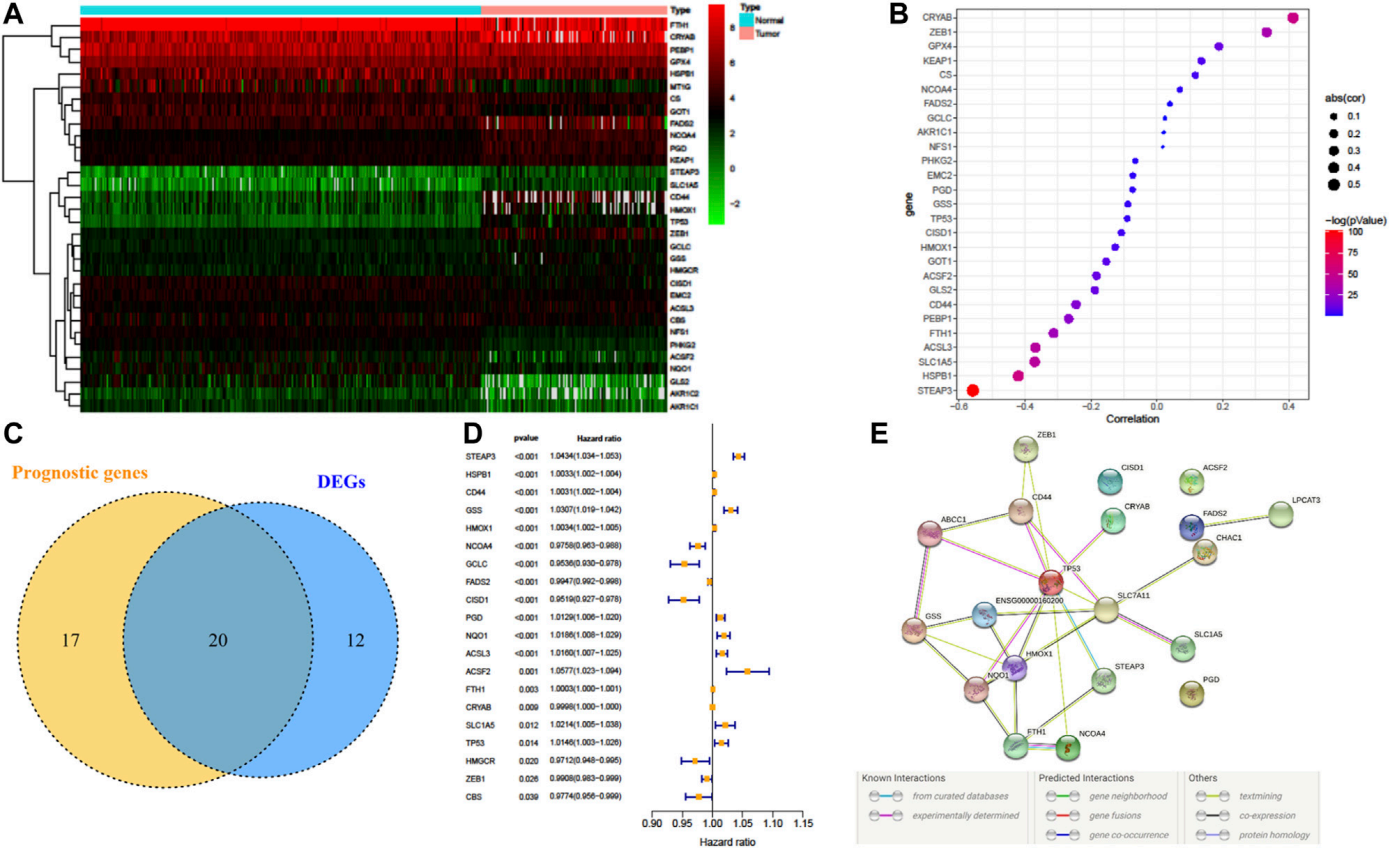
Dysregulation of ferroptosis-related genes (FRGs) and constructing a risk signature. **(A)** Heatmap of the differentially expressed FRGs between LGG and normal tissue. **(B)** Pearson’s correlation of FRGs between transcriptional expression and promoter methylation. **(C)** Venn diagram showing the overlapped gene number of differentially expressed FRGs and prognostic FRGs that correlated with OS. **(D)** Forest plot of hazard ratios demonstrating the prognostic value of overlapped FRGs. **(E)** PPI network indicating the interactions among the overlapped FRGs.

32 FRG expressions were identified to have a significant difference between LGG samples from TCGA and normal samples from GTEx (Figure 2A). Fifteen genes were upregulated in LGG, whereas another seventeen genes were downregulated in tumor. Since methylation of promotor plays a critical role in regulating gene expression, the effect of promotor DNA methylation on the gene expression of DE-FRGs was evaluated. As shown in Figures 2A,B, a significant negative correlation was found between gene expression and DNA methylation in five genes, including *FTH1*, *ACSL3*, *SLC1A5*, *HSPB1*, and *STEAP3*, whereas significant positive correlations were found in *CRYAB* and *ZEB1*. These results demonstrated that promoter DNA methylation might regulate the expression of FRGs in LGG with complexly regulative patterns.

### Construction of a Prognostic FRG Signature

**FIGURE 3:**
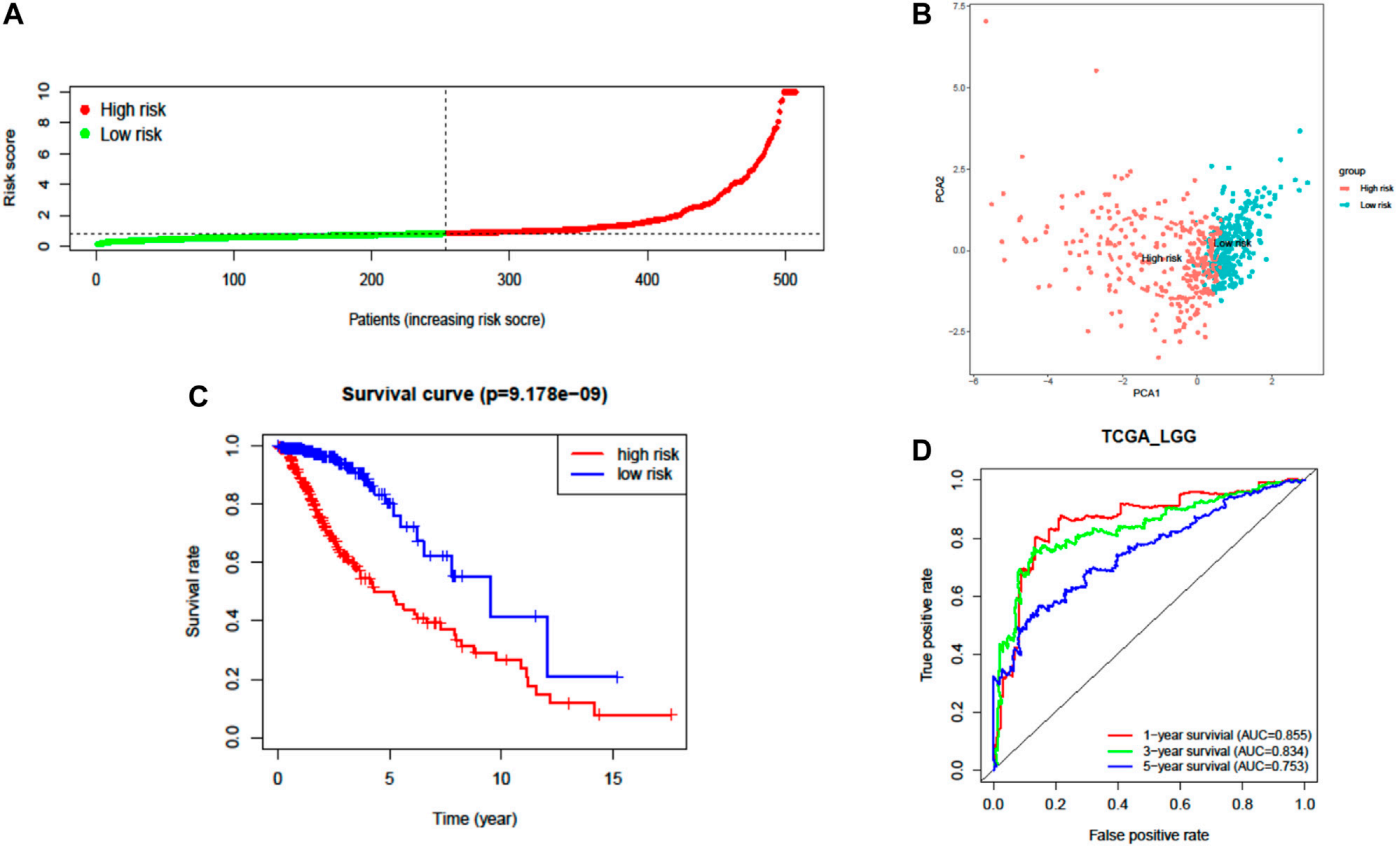
Prognostic analysis of the seven-FRG signature model in TCGA cohort. **(A)** Distribution and median value of the risk scores. **(B)** PCA plot of TCGA cohort. **(C)** Kaplan–Meier curves for the OS of patients in the high-risk group and low-risk group. **(D)** The AUC of time-dependent ROC curves verified the prognostic performance of the risk score.

A total of 37 FRGs were found for potent prognostic value (*p* < 0.05) through univariate Cox proportional hazard regression. 20 of them were differentially expressed between LGG and normal tissue (Figures 2C,D). The PPI network among these genes was constructed using the STRING online database, with parameters including a minimum required interaction score >0.4 (medium confidence). As shown in Figure 2E, *TP53*, *SLC7A11*, and *HMOX1* were hub genes for ferroptosis in LGG (Figure 2E). LASSO regression analysis was performed on the 20 overlapped genes, and nine genes were retained according to the optimal lambda value (Supplementary Figure S1). By using multivariate Cox regression analysis, a prognostic model based on seven genes was developed. The prognostic risk score was imputed as follows: Risk score = (0.0086 × expression level of *ACSL3*) + (−0.0190 × expression level of *CBS*) + (0.0013 × expression level of *CD44*) + (−0.0027 × expression level of *FADS2*) + (0.0017 × expression level of *HSPB1*) + (0.0104 × expression level of *PGD*) + (0.0271 × expression level of *STEAP3*). The 509 patients were classified into high- and low-risk groups according to the median value as cutoff (Figure 3A and Supplementary Figures S2A,C). The PCA plot showed the patients in two risk groups were distributed in discrete directions (Figure 3B). Kaplan–Meier (K–M) plots indicated patients with high risk scores had a significantly worse OS probability (*p* = 9.178e-9) (Figure 3C). To verify the diagnostic competence of the ferroptosis-related risk signature, the AUC for OS prediction was calculated. The AUC of the ROC curve reached 0.855 at 1 year, 0.834 at 3 years, and 0.753 at 5 years, which indicated that the risk score presented excellent performance on prognostic prediction of TCGA-LGG (Figure 3D). The false negative rate (FNR) of prognosis for OS at 1 , 3 , and 5 years was 0.077, 0.118, and 0.169, respectively (Supplementary Table S3).

### Validation of the Molecular Signature in CGGA-LGG Dataset

**FIGURE 4:**
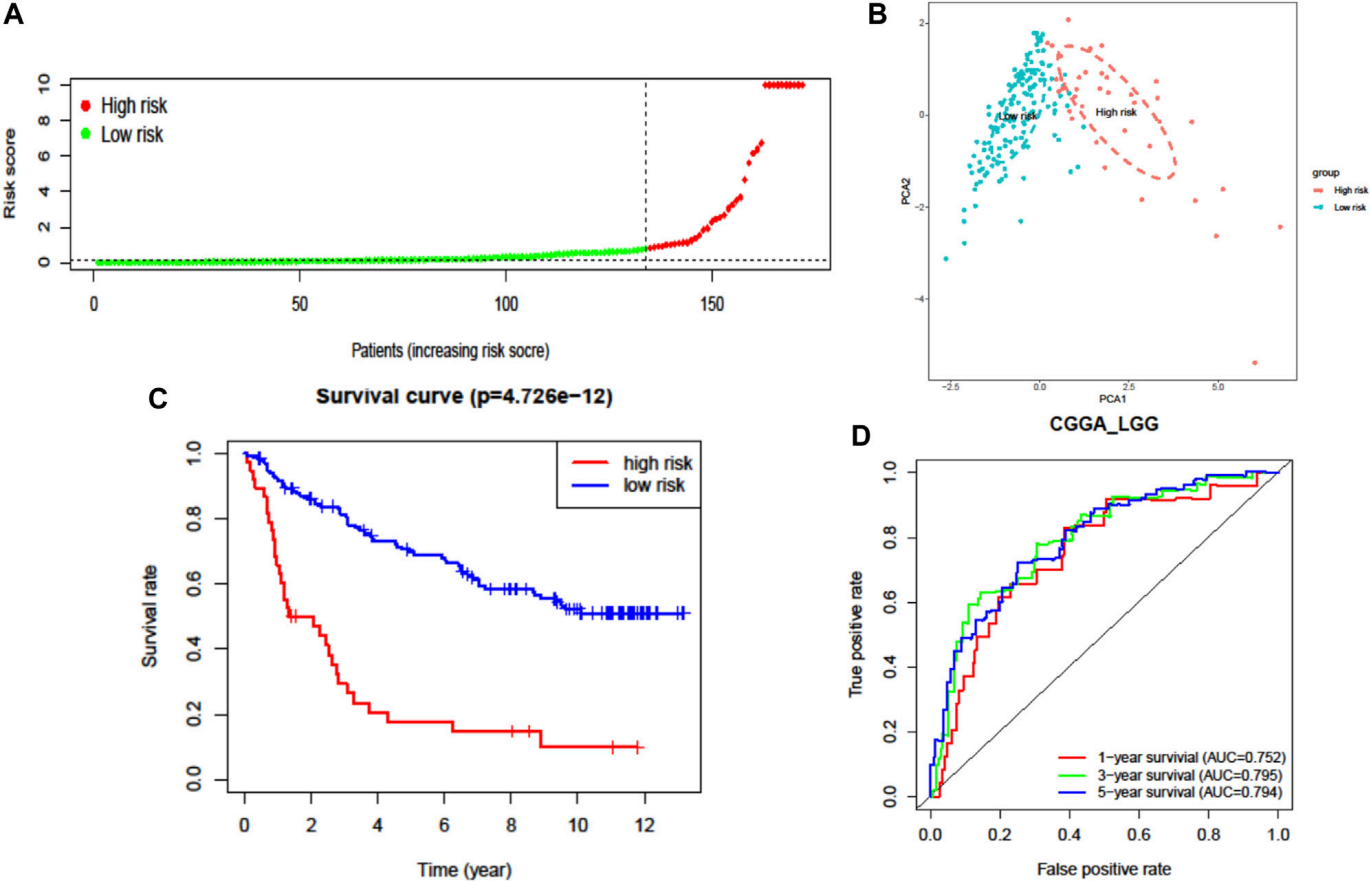
External validation of the seven-FRG signature model in the CGGA cohort (*n* = 170). **(A)** Distribution of the risk scores. **(B)** PCA plot of the CGGA cohort. **(C)** Kaplan–Meier curves for the OS of patients in the high-risk group and low-risk group. **(D)** The AUC of time-dependent ROC curves verified the prognostic performance of the risk score.

The prognostic capability of the FRG risk signature was externally validated by the CGGA dataset. The patients from the CGGA cohort were also divided into low- or high-risk groups by the same cutoff value as that in TCGA cohort (Figure 4A, Supplementary Figures S2B,D, and Supplementary Figure S3). Consistent with the above findings, PCA found that the patients in different risk groups were distributed in two directions (Figure 4B). Additionally, the K–M survival curves showed a significantly worse survival rate with high-risk patients (Figure 4C and Supplementary Figure S3A). The AUC for one-, three-, and five-year survival was 0.752, 0.795, and 0.794, respectively (Figure 4D). These results confirmed that the seven-FRG prognostic model presented good performance in prognostic prediction of LGG.

### Identification of the FRG Signature as an Independent Prognostic Factor

**FIGURE 5:**
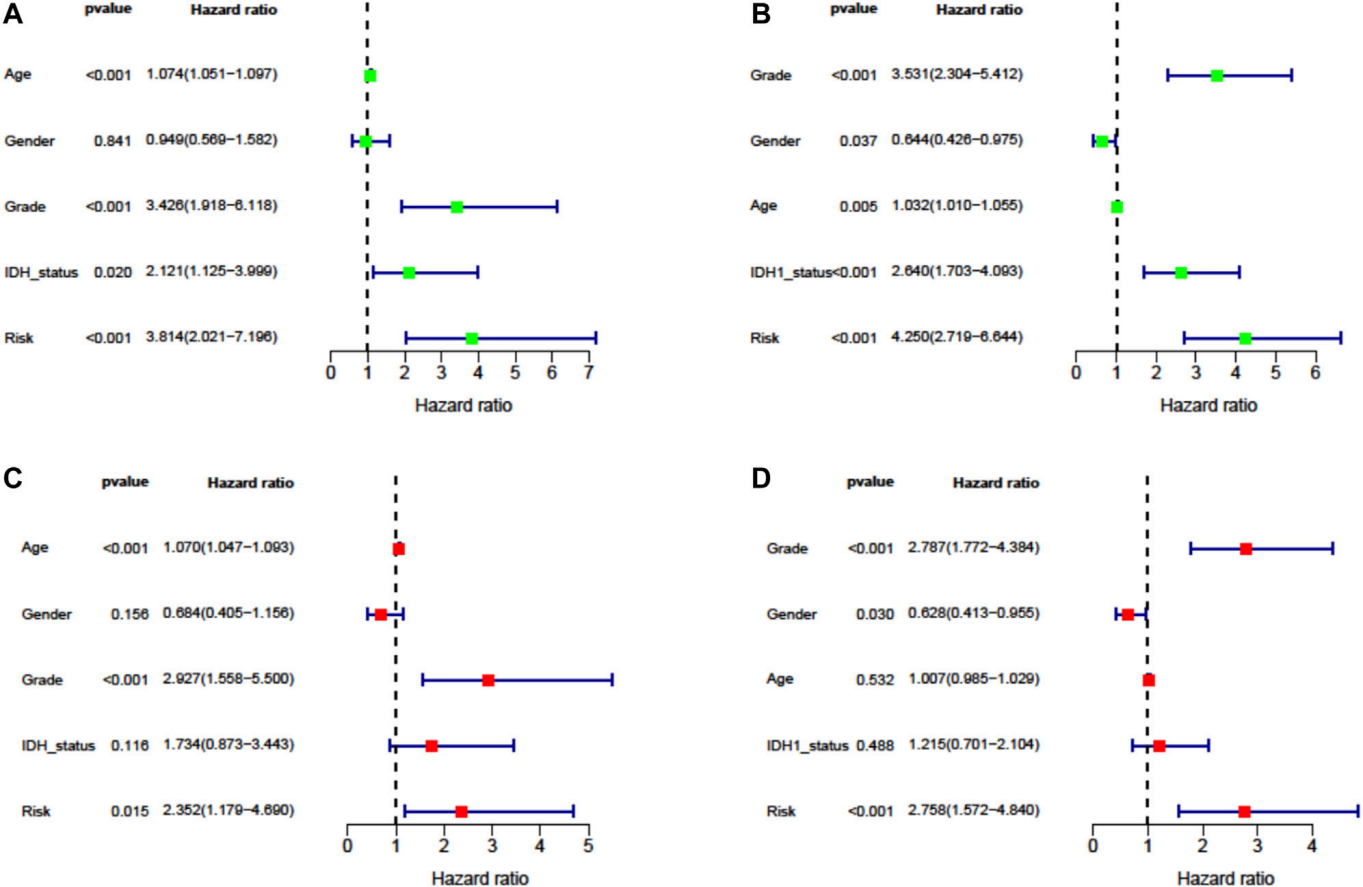
Cox proportion hazard regression for the OS of LGG. **(A, B)** Univariate Cox regression analysis in TCGA derivation cohort and the CGGA validation cohort. **(C, D)** Multivariate Cox regression analysis in TCGA derivation cohort and the CGGA validation cohort.

To evaluate the independent prognostic force of the signature, both the univariate and multivariate Cox proportion hazard regression models were applied. The risk score in univariate analysis was significantly correlated with OS in both TCGA and CGGA cohorts (HR = 3.814, 95% CI = 2.021–7.196, *p* < 0.001; HR = 4.250, 95% CI = 2.719–6.644, *p* < 0.001, respectively) (Figures 5A,B). The results from multivariate analysis found this signature was an independent prognostic factor for OS in TCGA-LGG (HR = 2.352, 95% CI = 1.179–4.690, *p* = 0.015) and CGGA-LGG (HR = 2.758, 95% CI = 1.572–4.840, *p* < 0.001) cohorts. These results confirmed the independent prognostic value of the seven-FRG–based prognostic signature for patients with LGG (Figures 5C,D).

### Construction and Verification of Nomogram

**FIGURE 6:**
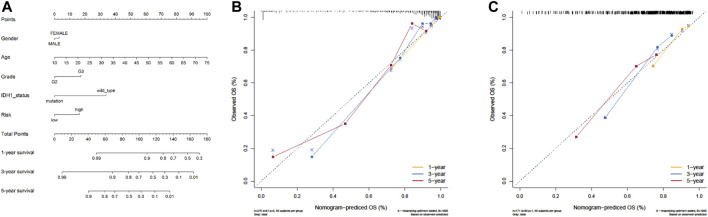
The nomogram can predict the survival probability in LGG. **(A)** A nomogram of TCGA cohort used to predict the OS. **(B, C)** Calibration curves used to predict the one-, three-, and five-year survival in TCGA derivation cohort and the CGGA validation cohort. The x-axis and y-axis represent the predicted and actual survival rates of the nomogram, respectively.

Next, by integrating the above risk signature with clinicopathological factors, including age, gender, grade, and IDH status, a nomogram was cautiously constructed for prediction of one-, three-, and five-year survival rates of LGG. As shown in Figure 6A, each level of factors was assigned one point, and a total point was calculated by adding up the points in each individual. Subsequently, the C-indexes of the nomogram for predicting OS were 0.87 (95% CI, 0.83–0.91) and 0.68 (95% CI, 0.63–0.73) in TCGA and CGGA cohorts, respectively. Calibration curves also showed good agreement between predictive and observational values at the probabilities of one-, three-, and five-year survival in TCGA and CGGA cohorts (Figures 6B,C). These results revealed that the nomogram signified good accuracy in predicting the one-, three-, and five-year survival of patients with LGG.

### Functional Annotation and Enrichment Analysis of the FRG Prognostic Signature

**FIGURE 7:**
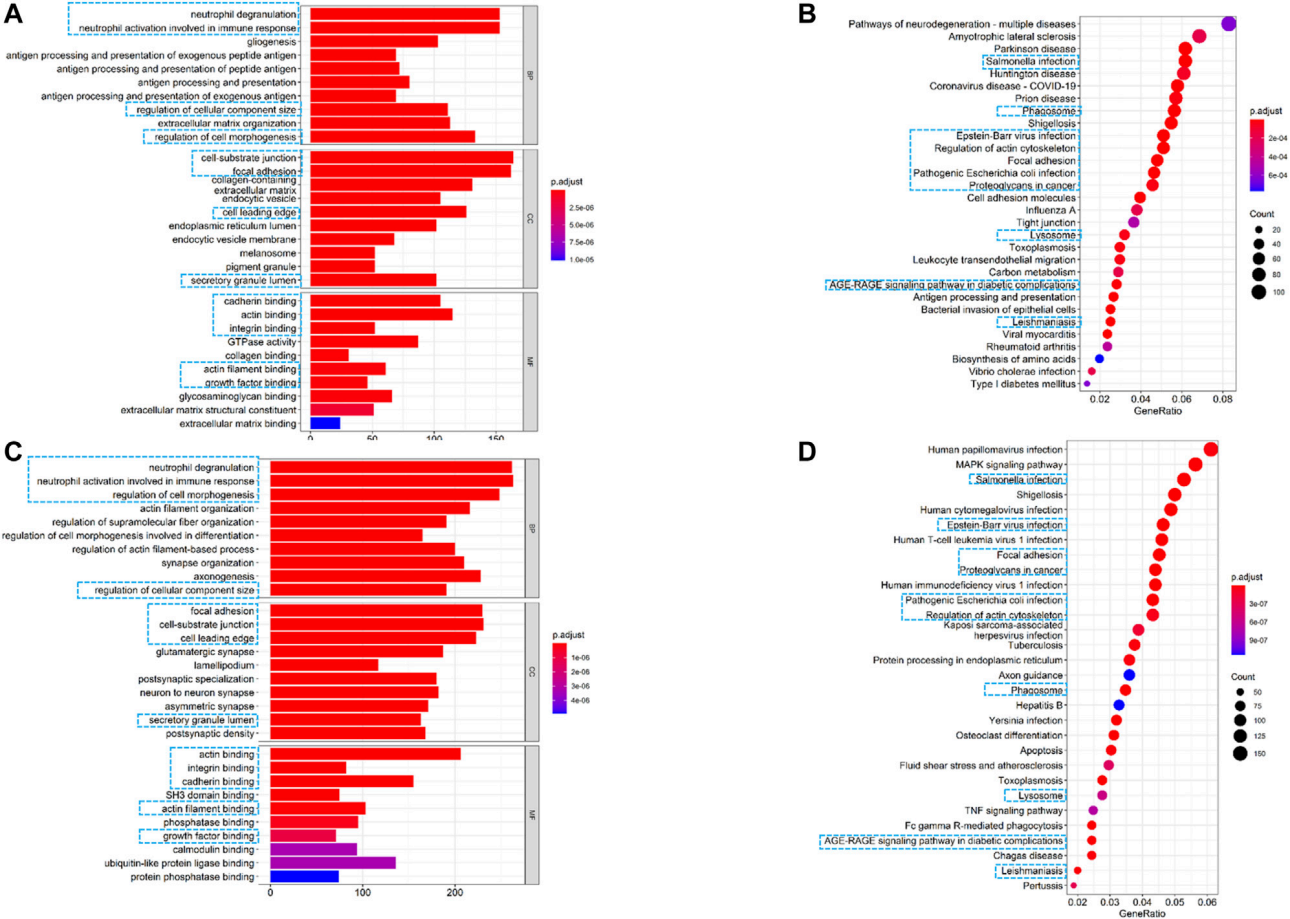
Altered functional characteristics related to the seven-FRG signature. **(A)** Representative results of GO in TCGA cohort. **(B)** KEGG analyses in TCGA cohort. **(C)** Representative results of GO in the CGGA cohort. **(D)** KEGG analyses in the CGGA cohort. The blue rectangles indicate the overlapped terms between the two cohorts.

To further elucidate the biological functions and pathways associated with the risk signature, we performed GO and KEGG pathway enrichment analyses based on differentially expressed genes (DEGs) between the high- and low-risk groups. As shown in Figures 7A,C, GO analysis showed 13 clusters sets were enriched in both TCGA and CGGA cohorts. For biological processes (BPs), DEGs were mainly enriched into immune-related terms, including neutrophil degranulation and neutrophil activation involved in immune response. For cellular components (CCs), cell-substrate junction, focal adhesion, cell leading edge, and secretory granule lumen were found to be enriched. With respect to molecular function (MF), differentially expressed genes were mainly enriched in actin binding, integrin binding, cadherin binding, actin filament binding, and growth factor binding. For the KEGG pathway, 10 out of top 30 pathways were enriched in both cohorts, and most of these enriched pathways were related to infections of bacterial or viral (Figures 7B,D). Interestingly, the term of focal adhesion, which was also reported closely related to the tumor immune microenvironment (TME) (Murphy et al., 2020), was enriched in both GO and KEGG analyses. These results revealed that the risk signature was correlated with the infection and immune-related pathway.

**FIGURE 8:**
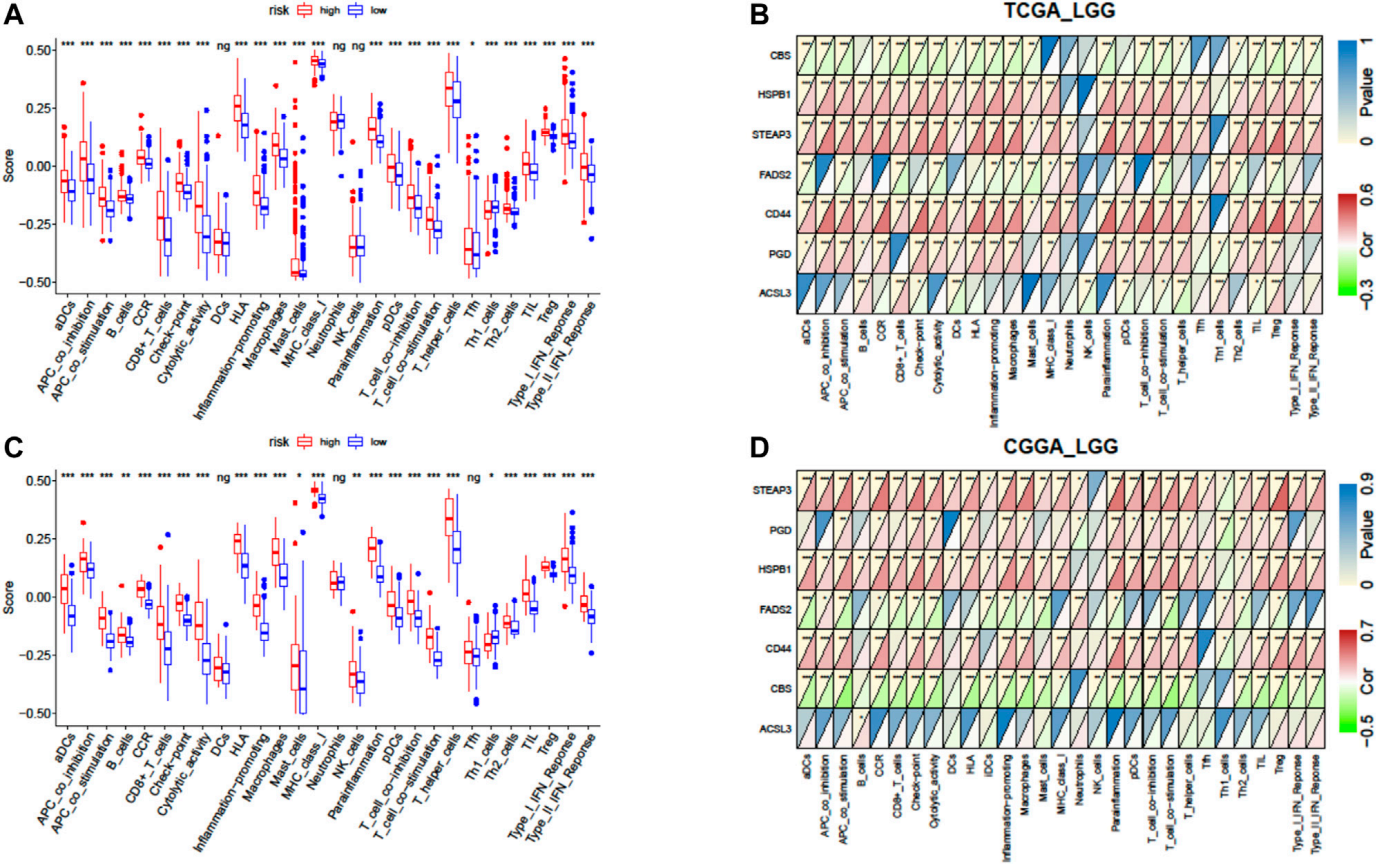
Correlation of the risk score with infiltrative immune cells. **(A)** Comparison of the ssGSEA scores between different risk groups in TCGA cohort. **(B)** Correlations between ssGSEA scores and risk scores in TCGA cohort. **(C)** Comparison of the ssGSEA scores between different risk groups in the CGGA cohort. **(D)** Correlations between ssGSEA scores and risk scores in the CGGA cohort. P values are shown as follows: ns, not significant; *p < 0.05; **p < 0.01; ***p < 0.001.

Furthermore, we quantified the enrichment scores (ESs) of diverse immune cell subtypes and related functions or pathways with ssGSEA to confirm the correlation between the risk signature and immune status. As shown by the boxplot in Figure 8A, in TCGA-LGG cohort, 25 out of 28 immune-related terms were significantly higher in the high-risk group (*p* < 0.05, Figure 8A), except DCs (dendritic cells), neutrophils, and NK cells (natural killer cells). Consistently, twenty-four immune-related terms were validated by the ICGC cohort, except Tfh cells (T follicular helper cells), while NK cells were significantly higher in the high-risk group (*p* < 0.01, Figure 8C).

We further investigated the association between expressions of the seven-gene signature and the immune status ES in detail by calculating Pearson’s correlation. The results showed that most of the seven genes were positively correlated with these immune cells and functions, but *CBS* (*cystathionine beta-synthase*) and *FADS2* (*fatty acid desaturase 2*) were negatively correlated with these immune terms in both cohorts (*p* < 0.05, Figures 8B,D). However, inconsistent correlations of acyl-CoA synthetase long-chain family member 3 (*ACSL3*) with immune characters were observed between TCGA and CGGA cohorts (Figures 8B,D).

## Discussion

LGG, generally acknowledged as benign tumor and universally prone to progressing into high-grade glioma, is the major cause of mortality for young adults (Hottinger et al., 2016; Picca et al., 2018). In recent years, some molecular characteristics were found to be useful for LGG classification, prognosis prediction, and treatment guidance (Takano et al., 2016). However, demand for a novel prognostic biomarker of LGG is still urgent.

As a newly defined form of programmed cell death, ferroptosis and its role in oncology have attracted increasing levels of attention from researchers. It is well known that ferroptosis is involved in cancer pathology as an inhibitor or promotor. But its effect is exerted depending on the tumor type, stage, and microenvironment (Daher et al., 2019; Dai et al., 2020). It was also demonstrated that FRGs play roles in glioma cell death and drug resistance (Wang Z. et al., 2018; Hu et al., 2020). A 19-FRG signature also showed high correlation with clinical outcomes of glioma (Liu et al., 2020). These studies indicated the potential role of FRGs in glioma.

In this study, we identified that there were 29 differentially expressed FRGs in LGG, and seven of these genes were significantly correlated with promotor methylation. We used univariate Cox analysis to screen the relations between 29 DE-FRGs and prognosis, and 20 genes were found to be related to the survival of LGG. Followed by LASSO and multivariate Cox regression analysis, we developed an efficient risk model which consisted of seven FRGs and divided LGG patients into low- and high-risk groups by the risk score. The predictive performance of this risk model was validated by K–M and ROC curve analyses in both TCGA and CGGA cohorts.

Some clinical characters, including age (≤40 years vs. >40 years), tumor grade (II vs. III), and IDH status (wild-type vs. mutation), have been well-practiced in prognostic analysis for LGG patients (Nabors et al., 2020). Multivariate Cox analysis showed that the risk group was an independent prognostic indicator as well. Furthermore, we used the risk score and other clinical characteristics to construct a nomogram prognosis model. The nomogram performed well in prediction of OS probability, which was validated by time-dependent calibration plots and showed satisfactory performance in both TCGA and CGGA cohorts. Therefore, what we established is a clinically applicable prediction model to assist individualized survival prediction of patients with LGG.

Among the seven prognostic FRGs, only *HSPB1* and *CBS* were downregulated in LGG. Previous reports identified that they are both negative regulators for ferroptosis (Sun et al., 2015; Wang L. et al., 2018). Higher *HSPB1* was found to contribute to glioma development (Ye et al., 2016), while *CBS* expression, consistent with our results, was reduced in glioma progression (Takano et al., 2014). *ACSL3*, a key regulator of lipid metabolism, is required for inducing ferroptosis resistance (Magtanong et al., 2019). The previous studies indicate that *ACSL3* overexpression was associated with worse clinical outcomes in patients with NSCLC and melanoma (Chen et al., 2016; Padanad et al., 2016); in contrast, high *ACSL3* expression predicted a better prognosis in ovarian cancer (Chen et al., 2016). *CD44* expression suppressed ferroptosis in cancer cells and might exert its function as a glioma promoter by increasing tumor cell invasion and proliferation, which therefore could be a promising therapeutic target (Mooney et al., 2016; Liu et al., 2019). *FADS2* is upregulated in glioblastoma, and its deficiency inhibited glioblastoma cell proliferation, which offers a potential novel therapeutic target (Affronti and Wellen, 2019). *PGD* has been reported to overexpress in a number of cancer types and to be associated with poor prognosis. In addition, the upregulated expression of *PGD* also performs a key role in the development of radiochemotherapeutic resistance in cancer (Yang et al., 2018; Sarfraz et al., 2020). Six-transmembrane epithelial antigen of prostate 3 (*STEAP3*) is a ferrireductase, which is vital for cellular iron uptake and homeostasis (Ohgami et al., 2005). A recent investigation suggested that *STEAP3* might exert its function as an oncogenic mediator in glioma progression (Han et al., 2018). In our results, five of these seven FRGs (*ACSL3*, *CD44*, *HSPB1*, *PGD*, and *STEAP3*) were associated with poor prognosis, while the remaining two genes (*CBS* and *FADS2*) were the opposite.

Based on the DEGs between the two risk groups, we performed GO and KEGG annotation and surprisingly found that the term of focal adhesion was enriched in both GO and KEGG analyses. Focal adhesion–related terms, such as “integrin and growth factor binding,” were also enriched, which were considered closely related to cancer cell metastasis and tumor microenvironment (Murphy et al., 2020). A previous report that the expression of focal adhesion kinase was affected by high intracellular iron oxide nanoparticle concentrations (Soenen et al., 2010) may partially explain the high correlation between focal adhesion and ferroptosis catalyzed by iron. Additionally, some immune-related biological processes and pathways such as neutrophil-related terms in GO and infection pathways in KEGG were also enriched. The previous study found the ferroptosis of immune cells during infection is advantageous for infectious agents (Matsushita et al., 2015), but the regulatory mechanism between ferroptosis and infectious pathways in cancer is still unknown. Emerging evidence suggests that ferroptosis is associated with tumor immunity (Stockwell and Jiang, 2019; Liu et al., 2020; Tang et al., 2020). Consistently, our results on ssGSEA also indicated that the ferroptosis-related signature might positively regulate immune signaling pathways. High- and low- risk score groups defined by our risk model exhibited distinct immune landscapes. Specifically, we found that the ESs of universal immune features were significantly higher in the high-risk group. Pearson’s correlation test further revealed the expressions of *CD44*, *HSPB1*, *PGD*, and *STEAP3* were positively correlated with the general 38 immune ESs, while the expression of *CBS* and *FADS2* had an inverse correlation. The coefficients of *CBS* and *FADS2* in the risk model were negative, and they, together with other genes’ expression, contributed to the positive correlations of risk scores with immune ESs. In the CGGA cohort, it should be pointed out that *ACSL3* expression did not significantly correlate with immune feature ESs except the B cell. Recent studies confirmed that CD8+ T cells are required for glioma growth, and patients with higher abundance of CD8+ T cells in the TME were more likely to benefit from immune checkpoint inhibitor treatment (Tumeh et al., 2014; Kane et al., 2020). Taken together, a positive correlation between the risk score of our FRG model and immune characteristics indicated that the risk score could be a potential predictive biomarker for immune checkpoint blockade and worthy of further investigation. Understanding the role of immune checkpoint inhibitors in combination with ferroptosis inducers for gliomas is urgent to improve the efficacy of immunotherapy.

However, there were some limitations in our study. Firstly, the data came from two datasets with limited sample size, and additional robust analyses (Wu and Ma, 2015; Ren et al., 2019) in large-scale independent cohorts are needed in the future. Secondly, the prognostic factors in the nomogram cannot include some important prognostic factors in surgical resection, radiation treatment, or chemotherapy. The collection of detailed clinical information from samples might help to overcome these shortcomings and assess the diagnostic efficiency of the nomogram in future studies. Finally, the biological mechanisms and association with immunotherapeutic effect of the prognostic signature are still unknown. Further study is required to extend the understanding of ferroptosis and promote novel therapeutic strategies for LGG patients.

## Conclusion

In summary, we developed a seven-FRG prognostic signature and nomogram which have good performance in the prediction of survival of LGG patients from TCGA and CGGA datasets. Our preliminary observation on correlations between FRGs and tumor immune features also suggested that further investigation of underlying mechanisms between ferroptotic regulation and tumor immunity in LGG is warranted.

## Data Availability

The data of lower-grade glioma in this study were downloaded from The Cancer Genome Atlas (TCGA) and the Chinese Glioma Genome Atlas (CGGA). The data of the normal brain tissues were downloaded from Genotype-Tissue Expression Database (GTEx).

## Abbreviations

AUC, area under the ROC curve; CGGA, Chinese Glioma Genome Atlas; FDR, false discovery rate; FRGs, ferroptosis-related genes; GO, Gene Ontology; GSEA, gene set enrichment analysis; GTEx, Genotype-Tissue Expression; C-index, Harrell’s concordance index; KEGG, Kyoto Encyclopedia of Genes and Genomes; K–M, Kaplan–Meier; LASSO, least absolute shrinkage and selection operator; LGG, lower-grade glioma; OS, overall survival; ROC, receiver-operating characteristic; TCGA, The Cancer Genome Atlas
