# Supplementary material for: Prognostic Model and Nomogram Construction Based on a Novel Ferroptosis-Related Gene Signature in Lower-Grade Glioma
Source: Front Genet. 2021 Nov 8;12:753680. doi: 10.3389/fgene.2021.753680 (PMC8606636; doi:10.3389/fgene.2021.753680)
Supplement: Supplementary file 3 [file Presentation1.PPTX]

## Slide 1
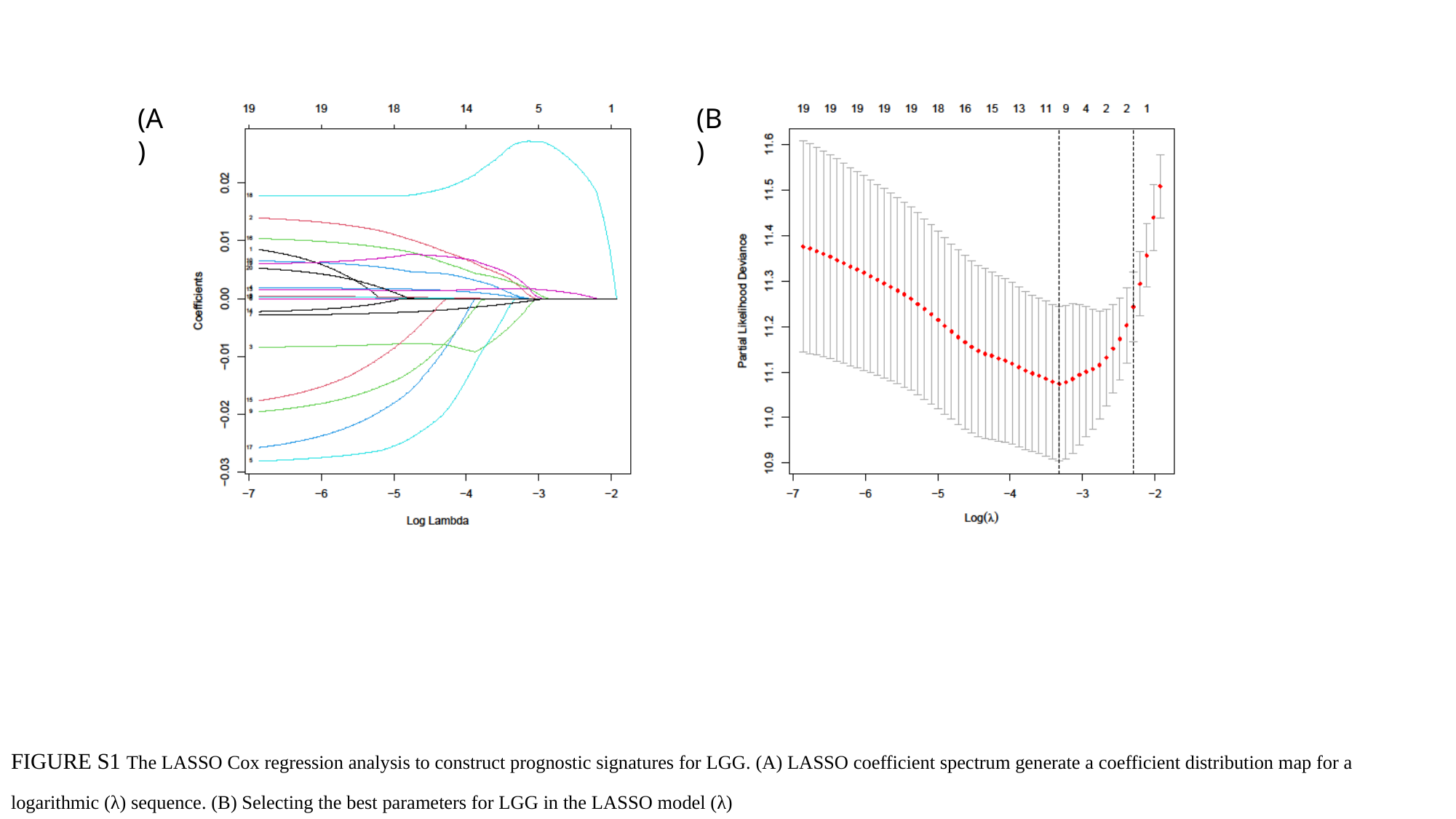

(A)
(B)
FIGURE S1 The LASSO Cox regression analysis to construct prognostic signatures for LGG. (A) LASSO coefficient spectrum generate a coefficient distribution map for a logarithmic (λ) sequence. (B) Selecting the best parameters for LGG in the LASSO model (λ)

## Slide 2
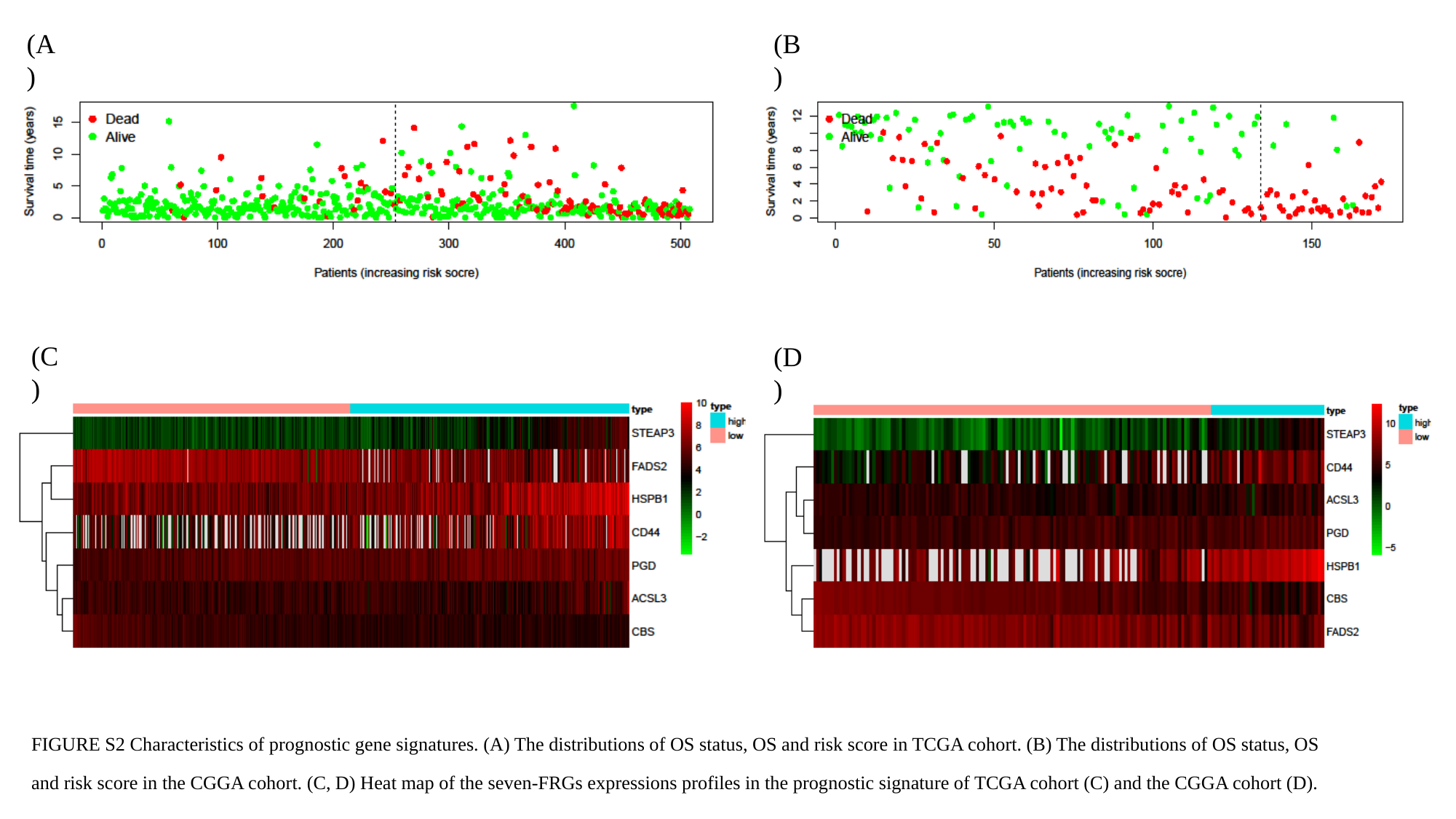

(A)
(B)
(C)
(D)
FIGURE S2 Characteristics of prognostic gene signatures. (A) The distributions of OS status, OS and risk score in TCGA cohort. (B) The distributions of OS status, OS and risk score in the CGGA cohort. (C, D) Heat map of the seven-FRGs expressions profiles in the prognostic signature of TCGA cohort (C) and the CGGA cohort (D).

## Slide 3
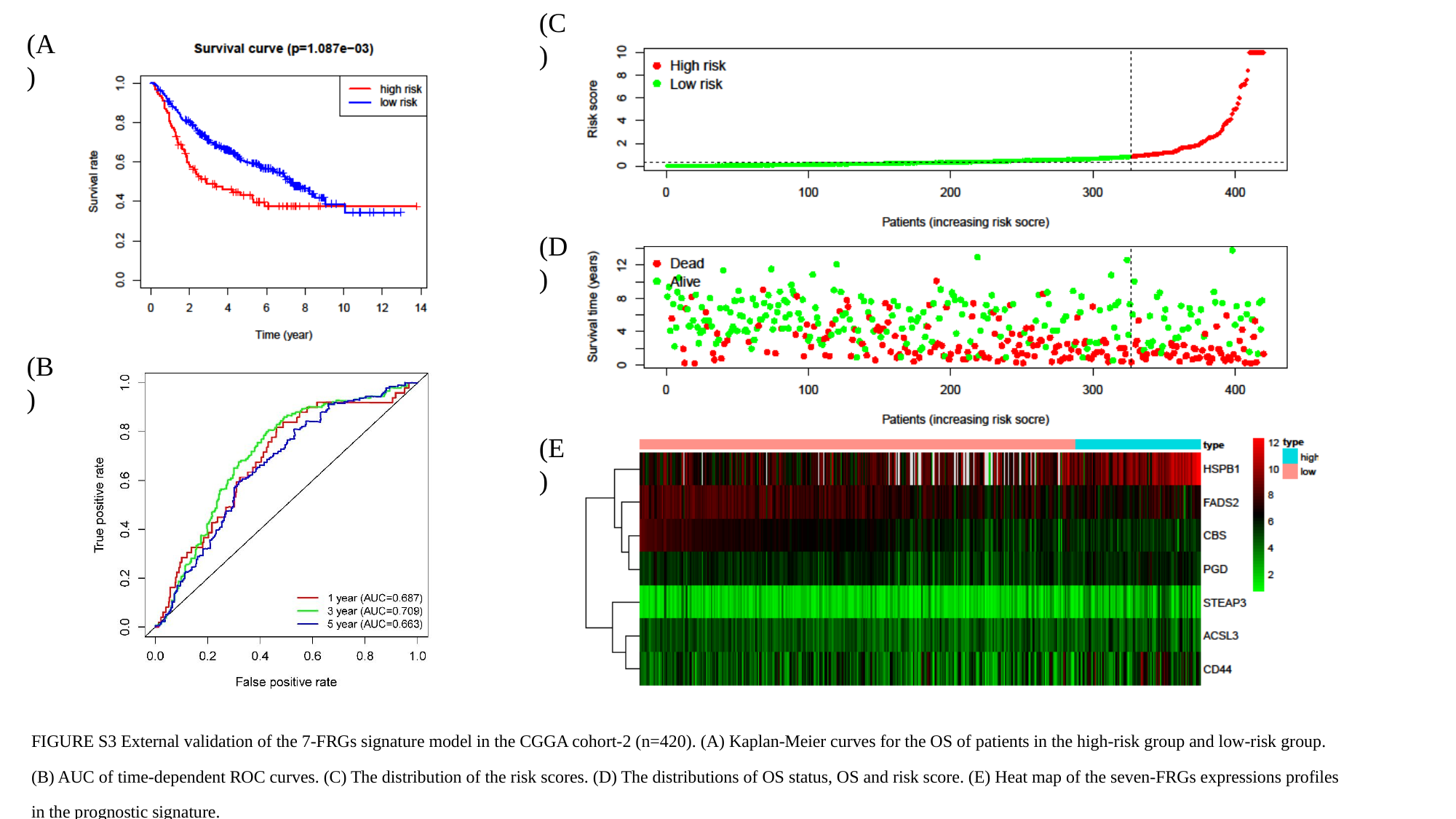

(C)
(A)
(D)
(B)
(E)
FIGURE S3 External validation of the 7-FRGs signature model in the CGGA cohort-2 (n=420). (A) Kaplan-Meier curves for the OS of patients in the high-risk group and low-risk group. (B) AUC of time-dependent ROC curves. (C) The distribution of the risk scores. (D) The distributions of OS status, OS and risk score. (E) Heat map of the seven-FRGs expressions profiles in the prognostic signature.
