# Supplementary material for: Prognostic Model and Nomogram Construction Based on a Novel Ferroptosis-Related Gene Signature in Lower-Grade Glioma
Source: Front Genet. 2021 Nov 8;12:753680. doi: 10.3389/fgene.2021.753680 (PMC8606636; doi:10.3389/fgene.2021.753680)
Supplement: Supplementary file 4 [file Table3.DOCX]

Table S3 False Negative Rate (FNR) of 7-FRGs prognostic model for survival

| Survival | FNR in TCGA cohort (%) |
| --- | --- |
| 1-year | 7.7 (2/26) |
| 3-year | 11.8 (8/68) |
| 5-year | 16.9 (14/83) |
